# Implementation of transbronchial lung cryobiopsy in a tertiary referral center for interstitial lung diseases: a cohort study on diagnostic yield, complications, and learning curves

**DOI:** 10.1186/s12890-021-01438-1

**Published:** 2021-02-25

**Authors:** Jesper Rømhild Davidsen, Inge Raadal Skov, Ida Guldbæk Louw, Christian B. Laursen

**Affiliations:** 1 Department of Respiratory Medicine, Odense University Hospital, Odense, Denmark; 2 Department of Respiratory Medicine, South Danish Center for Interstitial Lung Diseases (SCILS), Odense University Hospital, Kloevervaenget 2, 5000 Odense C, Denmark; 3 Odense Respiratory Research Unit (ODIN), Department of Clinical Research, University of Southern Denmark, Odense, Denmark; 4 Odense Patient Data Explorative Network, Odense University Hospital, Odense, Denmark

**Keywords:** Complications, Diagnostic yield, Interstitial lung diseases, Learning curves, Multidisciplinary discussion, Transbronchial lung cryobiopsy

## Abstract

**Background:**

Transbronchial lung cryobiopsy (TBLC) has been introduced as an alternative to surgical lung biopsy (SLB) in the diagnostics of interstitial lung diseases (ILD). Despite controversy on safety, TBLC is increasingly implemented in ILD centers with an apparent diagnostic yield comparable to SLB. The aim of this study was to assess TBLC implementation experiences from a tertiary Danish ILD center regarding diagnosis, complications, and learning curves for TBLC performance.

**Methods:**

TBLC was prospectively performed in a cohort of patients with unclassifiable ILD based on a preceding multidisciplinary clinical and radiological revision. TBLC was performed as an outpatient procedure with the patients in general anesthesia using a flexible bronchoscope with 1.9 or 2.4 mm cryoprobes. Learning curves for TBLC performance were calculated using cumulated sum (CUSUM) scores for diagnostic yield, pneumothorax, and bleeding.

**Results:**

From February 2017 to March 2020 141 patients (86 (61%) men, median age 69 years [IQR, 60–74 years]) had TBLC performed. A histological and confirmative diagnosis was made in 101 patients (75.2%) and 124 patients (87.9%, i.e. clinical diagnostic yield), respectively, in whom idiopathic interstitial pneumonias constituted the majority (67.3%) of the clinical diagnoses. We observed 2 deaths (1.4%) within 30 days of TBLC, but no procedure-related mortality or severe bleeding. Moderate bleeding occurred in 23 patients (16.3%), pneumothorax in 21 patients (14.9%) with only 14 patients (9.9%) requiring a pleural drain. Based on the CUSUM score analysis, the diagnostic yield obtained was satisfactory throughout the period.

**Conclusion:**

This study reports experiences of outpatient TBLC implementation in a tertiary referral ILD center from the largest investigated TBLC cohort in Scandinavia The diagnostic yield and prevalence of complications obtained by TBLC from this single center study on unclassifiable ILD support outpatient TBLC as a valuable and safe alternative to SLB to diagnose ILD in well-selected patients. The learning curves for TBLC were acceptable in the hands of experienced bronchoscopists.

## Introduction

Transbronchial lung cryobiopsy (TBLC) has been introduced as an alternative to surgical lung biopsy (SLB) in the diagnostics of interstitial lung diseases (ILD) and is increasingly implemented in ILD centers as an invasive outpatient procedure [1–3]. The indication for TBLC is to classify the specific ILD subtype in patients in whom the combination of medical history and information from a high-resolution computed tomography (HRCT) of the thorax is insufficient to obtain a confident clinical diagnosis [4, 5]. In such, the contribution of a histological diagnosis to determine a clinical ILD subtype is crucial in order to establish optimal treatment options and allow estimation of the overall prognosis of the disease [6, 7].

Though recommended standards for TBLC performance are now available [4, 8, 9], there are still diverging results regarding diagnostic yield and safety based on existing evidence from mainly retrospective studies accomplished in single centers with different organizational and set-up structures [10–16]. This, among other reasons, caused the recent international clinical guideline on idiopathic pulmonary fibrosis (IPF) to only endorse ILD centers with experience in TBLC to continue its use [7]. For many years SLB has been considered the golden standard for histological ILD diagnostics due to higher diagnostic yields of above 90% compared to approximately 83% in TBLC [7, 17, 18]. Despite controversy on the diagnostic validity and safety of TBLC [19], the recent prospective multicenter COLDICE study also favored continuing TBLC use due to a high agreement between SLB and TBLC in the assessment of ILD diagnoses [5]. In such, TBLC conducted at tertiary ILD referral centers seems as the diagnostic modality of choice with apparent diagnostic yields equaling SLB, but with a safety profile superior to SLB due to reduced risks of complications and mortality [17, 20–22].

This study aimed to assess outpatient TBLC experiences from a tertiary Danish ILD center with respect to diagnostic yield and complications. In addition, we assessed learning curves for TBLC performance.

## Methods

### Study design and setting

In this single center cohort study, we prospectively recorded data on patients who underwent TBLC following its implementation in March 2017 at the South Danish Center for Interstitial Lung Diseases (SCILS), Department of Respiratory Medicine, Odense University Hospital, Denmark, which serves as a tertiary ILD specialist center for the Region of Southern Denmark covering 1.223 million inhabitants (1st January 2020). Data collection ended on the 6th of March 2020.

### Study cohort

Based on a preceding multidisciplinary clinical and radiological revision eligible patients were 18 years or older with unclassifiable ILD with an HRCT ≤ 3 months, forced vital capacity (FVC) ≥ 50% of predicted value, diffusion capacity of the lung for carbon monoxide (DLCO) ≥ 40% of predicted value, an echocardiography ≤ 12 months with an estimated pulmonary systolic arterial pressure ≤ 40 mmHg, and a body mass index (BMI) ≤ 35 kg/m^2^ [9, 12]. Patients with a platelet count below 50,000 × 10^9^/L or a prothrombin time international normalized ratio (INR) above 1.5 were not eligible for TBLC.

### Procedure

Following state-of-the-art recommendations, TBLC was conducted by two experienced bronchoscopists (500+ bronchoscopies) as an outpatient procedure [8, 9]. Patients were intubated with a double luminal endobronchial tube in general anesthesia. Intravenous propofol, fentanyl, and rocuronium were used for the induction and maintenance of general anesthesia. A 4% lidocaine tracheal spray was used as local anesthetic in the upper trachea prior to intubation. Endotracheal intubation was performed using a bronchoscopy spiral tube (8.5 mm, Mediland, Rudersberg, Germany). Following the TBLC procedure sugammadex was used for reversal of neuromuscular blockade. TBLC was performed with a flexible bronchoscope (Olympus, Tokyo, Japan) using flexible cryoprobes (Erbe, Tubingen, Germany) with diameters of either 1.9 or 2.4 mm. The selection of the appropriate bronchopulmonary segment (BS) for TBLC was based on the individual patient’s most predominant interstitial lung abnormality (ILA) findings on HRCT. Every patient had a conventionally bronchoalveolar lavage (BAL) performed prior to TBLC and following a Fogarty balloon was inserted at the BS ostium and inflated to evaluate the appropriateness of its placement and its ability to “block” for potential distal bleeding secondary to TBLC. The cryoprobe was then introduced through the bronchoscope and into the selected BS. Using fluoroscopy, the placement of the tip of the cryoprobe was controlled as being placed approximately 10 mm from the thorax wall (visceral pleura) with a freezing time of 5 s [4]. When extracting the cryoprobe, the Fogarty balloon was synchronously inflated, and the TBLC samples were thawed in saline and following fixed in formalin. We performed at least 2 biopsies from 2 ipsilateral BS and slowly deflated the Fogarty balloon to observe whether bleeding emerged [23]. A focused lung ultrasound (FLUS) examination was used to identify potential iatrogenic pneumothorax (PTX) immediately after TBLC [24, 25]. The patient was observed for at least 2 h after TBLC, and a supplementary chest X-ray (CXR) was made to reveal late-onset PTX [9]. Drainage of PTX following the procedure were performed if one or more of the following were present:

1. FLUS with signs of PTX in the form of presence of lung point and placement of lung point indicating a large PTX (e.g. posterior to the midaxillary line)
2. CXR with signs of PTX with an intrapleural distance at the level of the hilum > 2 cm
3. FLUS or CXR with signs of PTX and a clinically unstable patient with signs of progressive respiratory failure

FLUS guided drainage with a pigtail catheter (Fr 7–16) was used as standard. The pigtail catheter was inserted either anteriorly or laterally depending on size of the PTX. In the case of treatment failure despite drainage with a pigtail catheter, a surgical drain was placed.

On a subsequent multidisciplinary team discussion (MDD) with presence of pulmonologists, radiologists, and pathologists a consensus diagnosis was reached on basis of the TBLC samples in conjunction with BAL, HRCT and other medical history of interest [7].

### Statistical analysis

The main outcomes were numbers and percentages of patients with a histological diagnosis, and the clinical MDD consensus diagnosis on basis of TBLC (diagnostic yield on basis of composite TBLC, BAL, clinical and radiological data). Secondary outcomes were numbers and percentages of complications secondary to TBLC as PTX, bleeding and mortality including associations between iatrogenic PTX and cryoprobe size. Bleeding severity was defined by as either minor (use of suction), moderate (use of Fogarty balloon and/or installation of cold saline), or severe (requiring transfusion and/or surgical intervention) [14, 17].

Categorical data are presented as numbers and prevalences. Continuous variables are presented as medians with interquartile ranges (IQR) (e.g., distribution of the different MDD consensus diagnoses, where the numerator represents the number of patients with a specific clinical MDD consensus diagnosis group and the denominator represents the total number of patients who underwent TBLC). All analyses were performed using Stata IC 16.1 (StataCorp, College Station, TX, USA).

Learning curves for both bronchoscopists were calculated using cumulated sum (CUSUM) scores for diagnostic yield, PTX, and bleeding. A successful procedure in terms of diagnostic yield was defined as a procedure in which the TBLC resulted in a diagnosis at the subsequent MDD. Complications such as PTX were defined as any patient who developed PTX following TBLC, irrespective of whether the patient received pleural drain insertion or was managed conservatively. Bleeding complications were defined as any bleeding occurring during the TBLC procedure. For the calculation of the CUSUM scores, acceptable and unacceptable diagnostic yield was defined as 90% and 80%, respectively. For both types of complications (i.a. PTX and bleeding), acceptable and unacceptable complication prevalences were defined as 10% and 20%, respectively. For the calculation of the predefined decision interval (H), α and β were both given values of 0.1, corresponding to a H_0_ and H_1_ of 2.71 [26]. When interpreting the learning curves decreasing values (downward deflection) indicate a successful procedure, whereas increasing values (upward deflection) indicate procedure failure in terms of obtaining a diagnosis or complications.

## Results

### Baseline characteristics

From the 1st of February 2017 to the 6th of March 2020, a total of 144 consecutive patients with unclassifiable ILD fulfilled the criteria for TBLC. However, one patient was omitted due to language problems, and for two patients TBLC was converted to other modalities, hereof mucosa biopsy in one patient with pulmonary sarcoidosis and endobronchial tumor removal in one patient with an endoluminal tumor (hamartoma). The remaining 141 patients underwent TBLC (61.0% males), with a median age of 69 years (IQR; 60–74 years)). The majority were current or past smokers (86 patients, 61.0%) with normal ventilation parameters with a minor restrictive pattern (median TLC of 77%; IQR 68.2–86.0%), moderately reduced DLCO (median DLCO of 57% (IQR; 40–64%)) and a 6-min walking distance (6MWD) of 435 m (IQR; 99–520 m). Baseline patient characteristics are given in Table 1.

**Table 1:** Baseline characteristics

Patient characteristics*	
Patients, N (%)	141 (100)
*Gender*	
Males, N (%)	86 (61.0)
Females, N (%)	55 (39.0)
Age, median (IQR)	69 (60–74)
*Smoking*	
Never smoker, N (%)	55 (39.0)
Past smoker, N (%)	63 (44.7)
Current smoker, N (%)	23 (16.3)
FEV1 (L), median (IQR)	2.5 (2.0–2.9)
FEV1 (% pred.), median (IQR)	89.0 (76.0–98.0)
FVC (L), median (IQR)	3.1 (2.6–3.7)
FVC (% pred.), median (IQR)	87.1 (77.8–102.0)
TLC (L), median (IQR)	4.8 (4.1–5.6)
TLC (% pred.), median (IQR)	77.0 (68.2–86.0)
DLCO (% pred.), median (IQR)	57.0 (40.0–64.0)
6MWD (m), median (IQR)	435 (99.0–520.0)

*DLCO* diffusion capacity of the lung for carbon monoxide, *FEV1* forced expiratory volume in 1 s, *FVC* forced vital capacity, *TLC* total lung capacity, *6MWD* 6-min walking distance

*Continuous data expressed as median with inter quartile range (IQR); categorical data as numbers (N) and percentages (%)

### Biopsy data and histological diagnoses

Among the 141 TBLC procedures performed, the 1.9 mm and 2.4 mm probe was used in 26 (18.4%) and 115 (81.6%) of the cases, respectively. Table 2 shows that all patients had 4 TBLCs performed with a median size of 5 mm (IQR; 5–7 mm). Every patient had biopsies obtained from the same lobe predominated by right lower lobe (88.7%), except from one patient in whom 2 biopsies were undertaken from both middle and right lower lobe. All TBLCs contained representative lung tissue. As presented in Table 3, the most common histological pattern was usual interstitial pneumonia (UIP) (39.0%) followed by non-specific interstitial pneumonitis (NSIP) (19.1%) and hypersensitivity pneumonitis (HP) (8.5%). A specific histological pattern was obtained in 106 patients (75.2%, i.e. histological yield). Non-diagnostic findings and normal lung tissue were found in 31 and 4 patients, respectively.

**Table 2:** Biopsy data, complications, and mortality

TBLC-procedure characteristics	
*Biopsy location*^*#*^	
ML, n (%)	2 (14.2)
RLL, n (%)	125 (88.7)
LLL, n (%)	15 (10.6)
*Bleeding*	141 (100.0)
Minor, n (%)	118 (83.7)
Moderate, n (%)	23 (16.3)
Severe, n (%)	0 (0.0)
Number of biopsies, median (IQR)	4 (4–4)
Biopsy size (mm), median (IQR)	5 (5–7)
Probe size 2.4 mm	5 (5–7)
Probe size 1.9 mm	5 (4–6)
*Pneumothorax, n (%)*	21 (14.9)
+ pleuradrain, n (%)	14 (9.9)
− pleuradrain, n (%)	7 (5.0)
Probe size 2.4 mm, n (%)	17 (81.0)
Probe size 1.9 mm, n (%)	4 (19.0)
Admission, n (%)*	21 (14.9)
*Mortality*	
Procedure related mortality, n (%)	0 (0.0)
30-days mortality, n/N (%)	2/141 (1.4)
90-days mortality, n/N (%)	2/131 (1.5)
1-year mortality, n/N (%)	4/99 (4.9)

*LLL* left lower lobe, *ML* middle lobe, *RLL* right lower lobe, *TBCL* transbronchial lung cryobiopsy

Continuous data expressed as median with inter quartile range (IQR); categorical data as numbers (N) and percentages (%)

^#^One patient had 2 TBCBs performed in both ML and RLL, respectively, why total N = 142

*Including observation due to e.g. bronchoscopy-related pain or cough

**Table 3:** Histological pattern on basis of TBLC

Histological characteristics	n (%)
UIP	55 (39.0)
UIP^#^	8 (5.7)
Possible UIP^§^	4 (2.8)
Probable UIP^#^	29 (20.6)
Indeterminate UIP^#^	14 (9.9)
Non-diagnostic	31 (22.0)
NSIP	27 (19.1)
Hypersensitivity pneumonitis	12 (8.5)
Normal lung tissue	4 (2.8)
Eosinophilic pneumonia	3 (2.1)
CTD-ILD	3 (2.1)
Sarcoidosis	2 (1.4)
Unspecific vasculitis	1 (0.7)
DI-ILD	1 (0.7)
DIP	1 (0.7)
Organizing pneumonia	1 (0.7)

*CTD-ILD* connective tissue disease interstitial lung disease, *DI-ILD* drug-induced interstitial lung disease, *DIP* desquamative interstitial pneumonia, *NSIP* non-specific interstitial pneumonia, *UIP* usual interstitial pneumonia, *TBLC* transbronchial lung cryobiopsy

^§^Histological classification according to clinical guideline from 2015 [40]

^#^Histological classification according to clinical guideline from 2018 [7]

### TBLC complications and mortality

Minor and moderate bleeding occurred in 118 (83.7%) and 23 (16.3%) patients, respectively. No cases of severe bleeding were observed. Twenty-one patients (14.9%) developed procedure related PTX in which 17 (81%) of the cases were associated to a probe size of 2.4 mm. Fourteen patients required pleura drainage and were subsequently hospitalized for an average of one day (median 1 day; interquartile range 1–3 days). However, a third of the PTX cases (7 patients, i.e. 5% of all patients) had only minor and asymptomatic PTXs which were managed conservatively. The TBLC was performed as an outpatient procedure, but 21 patients (14.9%) were admitted following PTX with pleura drain insertion and/or symptoms such as severe cough or chest pain needing further observation. Subsequent chart reviews following discharge identified readmission of 3 patients (2.1%), one due to pneumothorax development within the first week following TBLC whereas the other two patients had no clinical or radiological signs indicating relapse of pneumothorax. No procedure-related mortalities were observed. The 30-day and 90-day mortality was 1.4% (2/141 patients) and 1.5% (2/131 patients), respectively, as shown in Table 2.

### Clinical MDD consensus diagnoses

Available data on TBLC, BAL, HRCT and medical history were evaluated on a following MDD in which a clinical consensus diagnosis was obtained in 124 patients (87.9%), and independent of the probe size used (1.9 mm probe of 88.4%, and 2.4 mm probe of 87.8%). Among these, 3 patients had non-ILD diagnoses (i.e. asthma, emphysema, post-infection abnormalities), nonetheless, their tentative clinical diagnoses had all been suspected of HP at the time of referral for TBLC. The most frequent diagnoses were IPF (31.2%) followed by NSIP (23.4%) and HP (10.6%). Clinical MDD consensus after TBLC on specific diagnoses could not be reached in 14 patients categorized with unclassifiable ILD and in 3 patients fulfilling criteria for idiopathic pneumonia with autoimmune features (IPAF) (Table 4).

**Table 4:** Clinical MDD consensus diagnoses

Final clinical MDD diagnosis	n (%)
IPF	44 (31.2)
NSIP	33 (23.4)
Hypersensitivity pneumonitis	15 (10.6)
Unclassifiable ILD	14 (9.9)
CTD-related ILD	13 (9.2)
Eosinophilic pneumonia	4 (2.8)
IPAF	3 (2.1)
Sarcoidosis	3 (2.1)
Other^#^	3 (2.1)
Organizing pneumonia	2 (1.4)
DI-ILD^§^	2 (1.4)
Asbestosis	1 (0.7)
Bronchiolitis obliterans	1 (0.7)
DIP	1 (0.7)
RB-ILD	1 (0.7)
SR-ILD	1 (0.7)

*CTD-ILD* connective tissue disease interstitial lung disease, *DI-ILD* drug-induced interstitial lung disease, *DIP* desquamative interstitial pneumonia, *IPAF* interstitial pneumonia with autoimmune features, *IPF* idiopathic pulmonary fibrosis, *MDD* multidisciplinary team discussion, *NSIP* non-specific interstitial pneumonia, *RB-ILD* respiratory bronchiolitis interstitial lung disease, *SR-ILD* smoking-related interstitial lung disease

^#^The category “Other” includes three patients with asthma, emphysema, and post-pneumonic interstitial abnormalities, respectively

^§^DI-ILD secondary to two cases of methotrexate exposure

### Learning curves

**Fig. 1:**
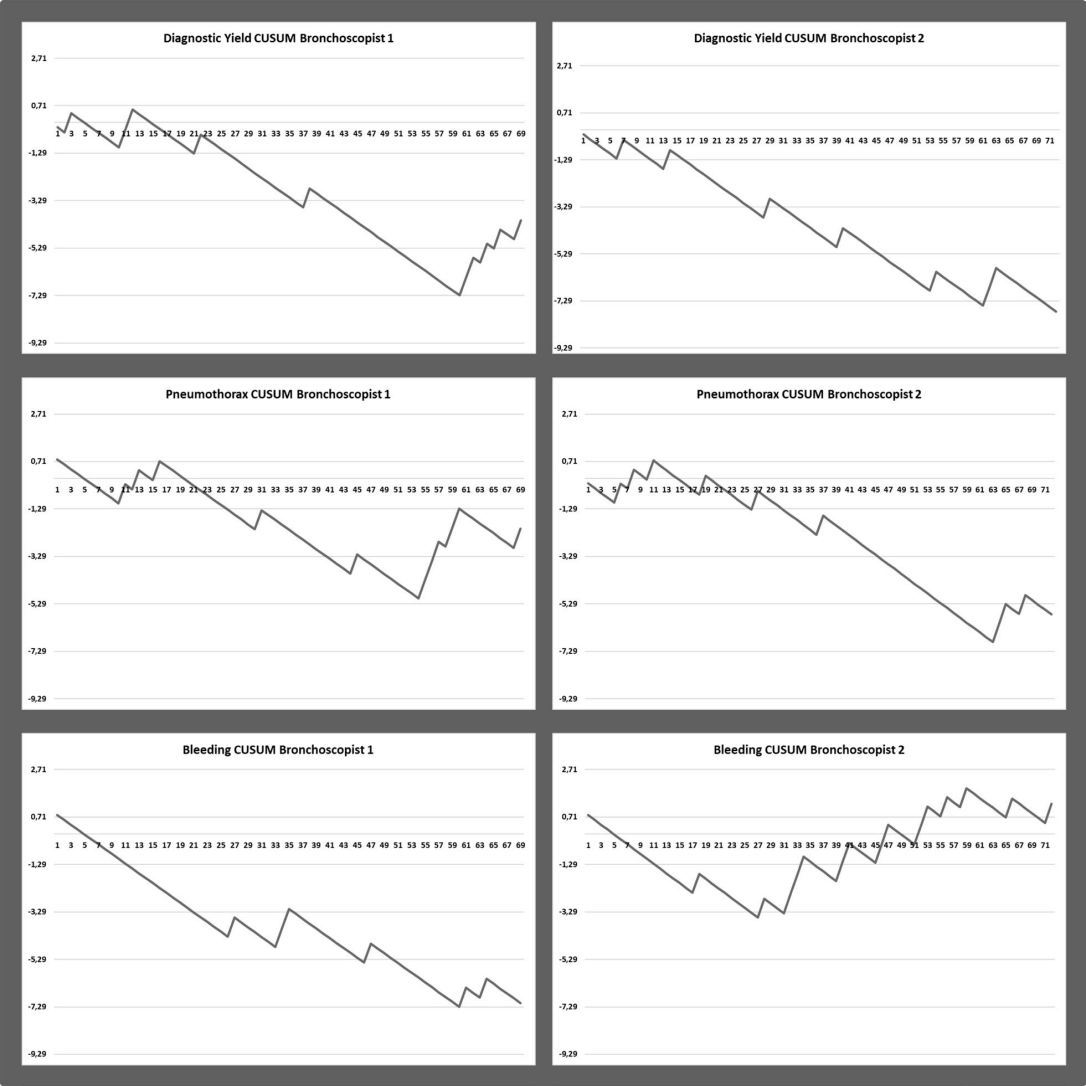
CUSUM curves for diagnostic yield, pneumothorax and bleeding for the two bronchoscopists. Decreasing values indicate a successful procedure whereas increasing values indicate procedure failure. None of the curves crossed the predefined decision interval of 2.71

Learning curves for the two bronchoscopists are presented in Fig. 1 and show CUSUM curves with a steady decline from the first procedures and an initial phase with no signs of difficulties in biopsy performance and of sufficient quality to obtain a diagnosis (i.e. diagnostic yield). Over time bronchoscopist #1 had some PTX complications, but less bleeding complications, whereas the opposite was present for bronchoscopist #2. For both bronchoscopists the number of complications were within the predefined acceptable prevalence of complications with none having CUSUM scores above the predefined decision interval.

## Discussion

To our knowledge, this study reports experiences of outpatient TBLC implementation in a tertiary referral ILD center from the largest investigated TBLC cohort in Scandinavia with only one preceding observational study [12]. Our results support previous findings to consider TBLC as a well-indicated modality in clinical practice for ILD diagnostics regarding diagnostic yield and safety [27].

Before the introduction of TBLC, the schism was to weigh pros and cons of transbronchial biopsy (TBB) and SLB in the diagnostics of ILD. The diagnostic yield of SLB has been reported above 90% [17, 18] compared to 30–50% for TBB [28], but at the risk of post-operative complications such as persisting pleural fistulas, chronic pain, and an increased procedure-related and 90-day mortality between 1.7 and 4.0% [29–31]. Retrospective findings on TBLC reveal adequate but lower diagnostic yields of up 83% compared to SLB [13, 15, 17], but is favorable in terms of reduced mortality of 0.3–1.7% compared to SLB of above 2.7% [3, 16, 17, 32]. On this basis, TBLC has been suggested as a first-choice diagnostic modality in ILD reserving SLB for only those cases in which TBLC samples have not contributed to a confident diagnosis [3, 17]. This approach is supported by recent findings from the prospective multicenter study (COLDICE) by Troy LK et al*.* in which 65 patients suspected of ILD underwent sequential TBLC and SLB [5]. The histological and diagnostic (i.e. MDD) agreement between TBLC and SLB was reported to be 70.8% and 76.9%, respectively, emphasizing TBLC to be a valid diagnostic tool for ILD when assuming the procedure conducted by experienced bronchoscopists [5, 27].

### Comparison to other studies

Due to the indications for TBLC and available recommendations for its performance [4, 8, 9], the patient characteristics of our cohort are quite comparable to cohorts from other ILD centers according to gender-, age-, lung physiology and smoking history [5, 12, 13, 16, 22, 33]. The typical candidate for TBLC was a 60+ year-old male with reduced DLCO, slightly restrictive lung function, however with preserved FEV1 and FVC. The histological patterns observed are in line with other study reports regarding distribution of NSIP, HP [12, 15], and UIP [22], but MDD obtained IPF diagnoses (31.2%) were more prevalent than otherwise reported [12, 16, 33]. The prevalence of unclassifiable ILD was lower (9.9%) than what is generally expected and observed in studies concerning ILD diagnostics [15, 34], and 2.1% fulfilled criteria for IPAF which is not a specific disease entity, but a proposed classification for patients with ILD and autoimmune features [35] (characteristics of IPAF patients is presented in Additional file 1: Table 1).

The histological and clinical diagnostic yields in our study were 75.2% and 87.9%, respectively. These values are slightly lower than in the study by Ravaglia et al*.* (87.8% and 90.1%) [22], but almost identical with results from Walscher et al*.* (73.4% and 83.5%) [15]. Addition of BAL had an impact on the final clinical MDD consensus diagnosis in 9 patients (6.4%) (Additional file 2: Table 2). However, our clinical diagnostic yield was considerably higher than reported in other smaller studies (50–74%) with similar TBLC settings and cohort characteristics [12, 16, 33]. The higher diagnostic yields in the study by Ravaglia et al*.* may be due to a larger number of patients investigated (N = 699) and TBLC performance in a center with longer TBLC experience [22].

In our ILD center, TBLC was planned as an outpatient procedure on stable patients, which is associated with a higher safety profile and being more cost-effective [5, 13, 36]. Except for one other Danish TBLC study [12], our reported diagnostic yields should be regarded with respect to TBLC performed with a flexible bronchoscope contrary to rigid bronchoscopes as used in most other studies. Thus, the majority of patients could leave the hospital within 6 h after TBLC, and only patients requiring further observation after e.g. PTX pleura drain insertion, instable pain or cough were admitted (14.9%). This set-up is different from other studies cited where TBLC involves elective admission of up to 3 days [3].

### Complications

Only 16.3% of the patients fulfilled the criteria for moderate bleeding, which somehow parallels other observations [12, 22], but was, however, quite lower than in the study by Ussavarungsi et al*.* [33]. We did not observe any case of severe bleeding related to TBLC.

PTX occurred in 14.9% of the patients. Though this prevalence seems higher than reported from systematic reviews [9, 17, 37], our PTX prevalence still lies within reported ranges from 0 to 26%. The 1:4 prevalence distribution of PTX according to 1.9 mm:2.4 mm probe equals to the 1:4 distribution in the probe size used. In such, this observation does not bring any of the probe sizes in favor in our cohort, though the 2.4 mm probe previously has been linked to an increased risk of PTX compared to the 1.9 mm probe [22]. However, due to small PTXs only two-thirds of the patients with PTX (i.e., 9.9% of all patients) required a pleura drain in our study, which is lower than documented in a study by Ravaglia et al*.* [17].

Based on the CUSUM score analysis, the diagnostic yield obtained by the two bronchoscopists seemed persistently satisfactory throughout the period, and especially with no obvious learning phase affection [26]. Similarly, the CUSUM curves assessing complications did not reveal any patterns of initial high prevalence of complications. After nearly 50 procedures the PTX complication prevalence increased for bronchoscopist #1. Conversely, bronchoscopist #2 had ongoing bleeding complications after approximately 30 procedures (max. moderate bleeding), which did not have any negative impact on diagnostic yield. For both bronchoscopists the prevalences of these complications were still below the predefined decision interval.

No TBLC procedure-related mortality was observed, but two patients (1.4%) died within 30 days due to an intracerebral bleeding and a presumed procedure-induced IPF exacerbation, respectively. Invasive procedures are well-known predictors of acute exacerbations in IPF, which was also the underlying diagnosis in the latter patient concluded on the subsequent MDD [36, 38]. At the time of TBLC performance, this patient did not show any clinical signs of acute exacerbation, in which TBLC would otherwise have been contraindicated [39]. The same two patients also contributed to the all cause 90-day mortality of 1.5% (2/131). Both the 30- and 90 days mortality in our study may seem higher than formerly reported [16, 17, 32], but do not exceed previously findings on this issue [3].

### Strengths and limitations

We find our cohort of unclassifiable ILD patients to be representative due to the comparable patient characteristics and ILD subtype distribution with other TBLC ILD cohorts. More composite factors may have contributed to increase the diagnostic yield to the best possible: TBLC was undertaken by the same two certified physicians according to international expert recommendations [9], compliance with the indication criteria for TBLC performance reduced the risk of selection bias and rendered comparable results with other TBLC cohorts, and not least a minimum 4 biopsies from 2 BS were conducted [23]. To diminish the risk of PTX and bleeding, TBLC was done under fluoroscopy, and in order to identify potential procedure related PTX, LUS was performed immediately after TBLC and a CXR after 2 h in line with expert statements [40].

The main limitation of the study was its observational design in a single center setting without any comparison of the diagnostic yields to a golden standard. Despite awareness of the patients smoking patterns, we did not examine whether concurrent comorbidities including BMI or medication status might have influenced the prevalence of complications. Furthermore, we restricted the cohort to the above-mentioned criteria including DLCO > 40%, whereas other centers have excluded patients with DLCO > 35% [22]. Due to this approach, we might have narrowed the number of patients eligible for TBLC. However, this difference may not have altered the histological diagnoses actually obtained by the chosen DLCO value.

The COLDICE study has provided evidence of TBLC as a valid diagnostic tool in ILD diagnostics and thus a relevant alternative to SLB [5]. This prompts speculations of whether the indication for TBLC in ILD diagnostics could be expanded to include patients not presently selectable for SLB and with worse DLCO and lung function than recommended in expert statements. It could be speculated that one way to increase the diagnostic accuracy regarding those patients not eligible for SLB due to bad constitution could be to apply TBLC to electromagnetic navigation bronchoscopy or to use smaller cryoprobes in order to obtain more peripheral (and hence more representative) interstitial lung biopsies [4]. However, the evidence on this point is still warranted in clinical practice.

## Conclusion

Our results are consistent with findings from other tertiary ILD centers with similar TBLC set-up presenting outpatient TBLC as a high-yield and safe procedure with low prevalence of complications and mortality, and hence suggesting TBLC to be the first-choice histological diagnostics in selected patients with unclassifiable ILD. The learning curves for TBLC in terms of complications and diagnostic yield were acceptable in the hands of experienced bronchoscopists.

## Supplementary Information

**Additional file 1: Supplementary Table 1**. Characteristics of three patients with IPAF diagnosis and their MDD conclusions.

**Additional file 2: Supplementary Table 2**. Nine cases in whom BAL was decisive in combination with TBLC on MDD.

## Data Availability

The datasets generated and/or used analysed during the current study are available from the corresponding author on reasonable request.

## Abbreviations

6MWD: 6-Min walking distance
ANA: Antinuclear antibody
Anti-CCP: Anti-cyclic citrullinated peptide
BAL: Bronchoalveolar lavage
BMI: Body mass index
BS: Bronchial segment
CD: Cluster of differentiation
CEP: Chronic eosinophil pneumonitis
CTD-ILD: Connective tissue disease interstitial lung disease
CUSUM: Cumulated sum scores
DLCO: Diffusion capacity of the lung for carbon monoxide
FEV1: Forced expiratory volume in one second
FVC: Forced vital capacity
GGO: Ground glass opacity
HRCT: High-resolution computed tomography
ILA: Interstitial lung abnormality
ILD: Interstitial lung disease
INR: Prothrombin time international normalized ratio
IPAF: Interstitial pneumonia with autoimmune features
IPF: Idiopathic pulmonary fibrosis
IQR: Inter quartile range
LLL: Left lower lobe
MDD: Multidisciplinary team discussion
ML: Middle lobe
MTX: Methotrexate
NSIP: Non-specific interstitial pneumonia
OP: Organizing pneumonia
PTX: Pneumothorax
RA-ILD: Rheumatoid arthritis interstitial lung disease
RB-ILD: Respiratory bronchiolitis interstitial lung disease
RF: Rheumatoid factor
RLL: Right lower lobe
SCILS: South Danish Center for Interstitial Lung Diseases
SLB: Surgical lung biopsy
TBLC: Transbronchial lung cryobiopsy
UIP: Usual interstitial pneumonia
