# Supplementary material for: Implementation of transbronchial lung cryobiopsy in a tertiary referral center for interstitial lung diseases: a cohort study on diagnostic yield, complications, and learning curves
Source: BMC Pulm Med. 2021 Feb 25;21:67. doi: 10.1186/s12890-021-01438-1 (PMC7908747; doi:10.1186/s12890-021-01438-1)
Supplement: Supplementary file 1 — Additional file 1: Supplementary Table 1. Characteristics of three patients with IPAF diagnosis and their MDD conclusions. [file 12890_2021_1438_MOESM1_ESM.docx]

**Title**

Implementation of transbronchial lung cryobiopsy in a tertiary referral center for interstitial lung diseases – a cohort study on diagnostic yield, complications, and learning curves

**Short Title**

Experiences from transbronchial lung cryobiopsy

**Authors and affiliations**

Jesper Rømhild Davidsen^a,b,c,d^, MD, PhD; Inge Raadal Skov^a,c^, MD; Ida Guldbæk Louw^a^, MD; Christian B. Laursen^a,b,c^, MD, PhD

^a^ Department of Respiratory Medicine, Odense University Hospital, Odense, Denmark

^b^ South Danish Center for Interstitial Lung Diseases (SCILS), Odense University Hospital, Odense, Denmark

^c^ Odense Respiratory Research Unit (ODIN), Department of Clinical Research, University of Southern Denmark, Denmark

^d^ Odense Patient data Explorative Network, Odense University Hospital, Odense, Denmark

***Corresponding Author**

Jesper Rømhild Davidsen, Associate Professor, MD, PhD

South Danish Center for Interstitial Lung Diseases (SCILS)

Department of Respiratory Medicine

Odense University Hospital

Kloevervaenget 2, DK – 5000 Odense C, Denmark

Tel: +45 21571292

Fax: +45 66124305

E-mail: jesper.roemhild.davidsen@rsyd.dk

**Supplementary Table 1:** Characteristics of three patients with IPAF diagnosis and their MDD conclusions.

| **Age and gender at TBLC** | **Paraclinical information** | **HRCT** | **Supplemetal information** | **BAL** | **TBLC** | **MDD**  **consensus**  **diagnosis**  **after TBLC** | **Latest**  **follow-up**  **status** |
| --- | --- | --- | --- | --- | --- | --- | --- |
| *Years*  *(M / F)* | *Autoimmunology and*  *lung physiology* | *Findings* | *Rheumatological evaluation* | *Cytological conclusion* | *Histological conclusion* | *Based on composite clinic-radiological-pathological findings* | *Based on ongoing composite*  *clinic-radiological-pathological-biochemical findings* |
| 70  (F) | - RF > 300 x 10^3^ IU/L - Anti-CCP 216 AU/mL - FEV1 1.15 L (54%) - FVC 1.63 L (60%) - DLCO 58% | Diffuse subpleural nodules with septal thickening in basal lung parts and mosaic attenuation without signs of airtrapping. Alternative diagnosis. | No clinical sign of RA or other CTDs. | Not representative. | Normal alveolar tissue without intraalveolar inflammation or fibrotic remodeling. Minor unspecific interstitial inflammation without representation of lymphocytic granulocytes. | IPAF. | - 21 months from TBLC - No interest in further invasive procedures. - Still positive RF. - Still positive anti-CCP. - Still no clinical signs of CTD. - Stable lung physiology and radiology. - Minor courses of Prednisolone due to episodes of increasing dyspnea. - Regularly 6 months clinical follow-up including lung physiology, autoimmunology and yearly HRCT. |
| 63  (F) | - RF 277 x 10^3^ IU/L - Anti-CCP 94 AU/mL - FEV1 1.42 L (66%) - FVC 1.76 L (69%) - DLCO 42% | Probable UIP. | After MDD, no clinical sign of RA or other CTDs. | Not representative. | TBLCs with heterogenic and fibrotic bronchiolocentric remodeling and chronic inflammation. No fibroblast foci or OP. Overall follicular inflammation with fibrotic remodeling. | IPAF with suspicion on underlying RA. | - 9 months from TBLC. - At 6 months follow-up, the patient had still positive IgM-RF and anti-CCP, but also evolved symptoms consistent with arthritis. - In such, a new rheumatological evaluation confirmed RA with pulmonary manifestation in form of RA-ILD. - The patient started MTX and Prednisolone treatment. - Half-yearly clinical follow-up including lung physiology and yearly HRCT in combination with rheumatological follow-up. |
| 61  (F) | - ANA >1:320 titer - FEV1 1.28 L (68%) - FVC 2.40 L (96%) - DLCO 61% | Probable UIP. | No clinical sign of RA or other CTDs. | Not representative due to bleeding. | Overall normal alveolar tissue with only minor unspecific inflammation. | IPAF. | - 9 months from TBLC. - At 6 months follow-up, the patient had still positive ANA. - Stable lung physiology and radiology. - Half-yearly clinical follow-up including lung physiology and yearly HRCT in combination with rheumatological follow-up. |

Abbreviations: ANA = antinuclear antibody, anti-CCP = anti-cyclic citrullinated peptide, BAL = bronchoalveolar lavage, CTD-ILD = connective tissue disease interstitial lung disease, DLCO = diffusion capacity of the lung for carbon monoxide, FEV1 = forced expiratory volume in one second, FVC = forced vital capacity, GGO = ground glass opacity, HRCT = high-resolution computed tomography, IPAF = interstitial pneumonia with autoimmune features, IPF = idiopathic pulmonary fibrosis, LLL = left lower lobe, MDD = multidisciplinary team discussion, ML = middle lobe, MTX = methotrexate, NSIP = non-specific interstitial pneumonia, OP = organizing pneumonia, RA-ILD = rheumatoid arthritis interstitial lung disease, RF = rheumatoid factor, RLL = right lower lobe, TBLC = transbronchial lung cryobiopsy, UIP = usual interstitial pneumonia.
