# Supplementary material for: Implementation of transbronchial lung cryobiopsy in a tertiary referral center for interstitial lung diseases: a cohort study on diagnostic yield, complications, and learning curves
Source: BMC Pulm Med. 2021 Feb 25;21:67. doi: 10.1186/s12890-021-01438-1 (PMC7908747; doi:10.1186/s12890-021-01438-1)
Supplement: Supplementary file 2 — Additional file 2: Supplementary Table 2. Nine cases in whom BAL was decisive in combination with TBLC on MDD. [file 12890_2021_1438_MOESM2_ESM.docx]

**Title**

Implementation of transbronchial lung cryobiopsy in a tertiary referral center for interstitial lung diseases – a cohort study on diagnostic yield, complications, and learning curves

**Short Title**

Experiences from transbronchial lung cryobiopsy

**Authors and affiliations**

Jesper Rømhild Davidsen^a,b,c,d^, MD, PhD; Inge Raadal Skov^a,c^, MD; Ida Guldbæk Louw^a^, MD; Christian B. Laursen^a,b,c^, MD, PhD

^a^ Department of Respiratory Medicine, Odense University Hospital, Odense, Denmark

^b^ South Danish Center for Interstitial Lung Diseases (SCILS), Odense University Hospital, Odense, Denmark

^c^ Odense Respiratory Research Unit (ODIN), Department of Clinical Research, University of Southern Denmark, Denmark

^d^ Odense Patient data Explorative Network, Odense University Hospital, Odense, Denmark

***Corresponding Author**

Jesper Rømhild Davidsen, Associate Professor, MD, PhD

South Danish Center for Interstitial Lung Diseases (SCILS)

Department of Respiratory Medicine

Odense University Hospital

Kloevervaenget 2, DK – 5000 Odense C, Denmark

Tel: +45 21571292

Fax: +45 66124305

E-mail: jesper.roemhild.davidsen@rsyd.dk

**Supplementary Table 2:** Nine cases in whom BAL was decisive in combination with TBLC on MDD.

| **Age and gender at BAL and TBLC** | **Supplemental information** | **BAL / TBLC** | **BAL** | **TBLC** | **MDD consensus diagnosis** |
| --- | --- | --- | --- | --- | --- |
| *Years*  *(M / F)* | *Considerable clinical or radiological information* | *Site* | *Cytological conclusion* | *Histological conclusion* | *Based on composite clinic-radiological-pathological findings* |
| 70  (F) | RA. | ML / RLL | Total cell number of 18.4 x 10^6^ cells/100 mL. Lymphocytosis of 65% with CD4/CD8-ratio 5.7. Neutrophil granulocytes of 2%, no eosinophil granulocytes. | Peribronchiolar and chronic inflammation with OP and interstitial fibrosis. | CTD-ILD  (RA-ILD) |
| 72  (M) | HRCT with indeterminate UIP. | ML / RLL | Total cell number of 3.1 x 10^6^ cells/100 mL. Lymphocytosis of 21% with CD4/CD8-ratio 2.7. No neutrophil- or eosinophil granulocytes. | Indeterminate UIP. | NSIP |
| 67  (F) | RA. | ML / RLL | Total cell number of 13.2 x 10^6^ cells/100 mL. Lymphocytosis of 33% with CD4/CD8-ratio 2.7. No neutrophil- or eosinophil granulocytes. | Cell rich inflammation with predominantly peribronchiolar lymphocytic folliculitis and minor interstitial fibrosis. | CTD-ILD  (RA-ILD) |
| 72  (F) | HRCT with probable UIP. | ML / RLL | Total cell number of 8.7 x 10^6^ cells/100 mL. Lymphocytosis of 22% with CD4/CD8-ratio 1.5. Neutrophil granulocytes of 4%, no eosinophil granulocytes. | Indeterminate UIP and places with minor OP. | NSIP |
| 47  (F) | Ex-smoker with 26 pack-years. HRCT with minor apical centrilobular GGO. | ML / RLL | Total cell number of 15.7 x 10^6^ cells/100 mL. 90% macrophages with hyperpigmentation. Neutrophil granulocytes of 2%, eosinophil granulocytes of 3%, lymphocytes of 3%. | Focal remodeling with slightly septal thickening. Unspecific findings. | RB-ILD |
| 58  (M) | HRCT with apical GGO, subpleural consolidating infiltrates with air bronchograms, and centrilobular emphysema. Persistent blood eosinophilia of 0.8-1.0 x 10^9^ cells /L. | ML / RLL | Total cell number of 10.4 x 10^6^ cells/100 mL. 90% macrophages with hyperpigmentation. Neutrophil granulocytes of 7%, eosinophil granulocytes of 7%, lymphocytes of 2%. | Probable UIP. | CEP |
| 72  (M) | HRCT with indeterminate UIP. Ex-smoker with 21 pack-years. | ML / RLL | Total cell number of 9.1 x 10^6^ cells/100 mL. Neutrophil granulocytes of 2%, eosinophil granulocytes of 14%, lymphocytes of 13%. | Indeterminate UIP. | IPF  (low confidence) |
| 69  (M) | HRCT with diffuse mosaic attenuation and GGO and centrilobular GGO micronodules. No obvious exposures. | ML / RLL | Total cell number of 4.7 x 10^6^ cells/100 mL. Lymphocytosis of 76% with CD4/CD8-ratio 3.7. No neutrophil- or eosinophil granulocytes. | Alveolar inflammation with alveolar wall thickening, focal OP, poorly formed non-necrotizing granulomas, and patchy distribution of cholesterol crystals. | HP |
| 74  (M) | HRCT with indeterminate UIP. Non-smoker. | ML / RLL | Total cell number of 13.5 x 10^6^ cells/100 mL. Neutrophil granulocytes of 16%, eosinophil granulocytes of 12%, lymphocytes of 7%. | Predominantly lymphocytic inflammation with focal areas of OP, and some alveolar wall thickening. No fibroblast foci or fibrosis. “Cellular NSIP pattern.” | NSIP |

Abbreviations: BAL = bronchoalveolar lavage, CD = cluster of differentiation, CEP = chronic eosinophil pneumonitis, CTD-ILD = connective tissue disease interstitial lung disease, F = female, GGO = ground glass opacity, HRCT = high-resolution computed tomography, IPF = idiopathic pulmonary fibrosis, M = male, MDD = multidisciplinary team discussion, ML = middle lobe, NSIP = non-specific interstitial pneumonia, OP = organizing pneumonia, RA-ILD = rheumatoid arthritis interstitial lung disease, RB-ILD = respiratory bronchiolitis interstitial lung disease, RLL = right lower lobe, SR-ILD = smoking-related interstitial lung disease, TBLC = transbronchial lung cryobiopsy, UIP = usual interstitial pneumonia.
